# Genome-wide *in silico *screen for CCCH-type zinc finger proteins of *Trypanosoma brucei*, *Trypanosoma cruzi *and *Leishmania major*

**DOI:** 10.1186/1471-2164-11-283

**Published:** 2010-05-05

**Authors:** Susanne Kramer, Nicola C Kimblin, Mark Carrington

**Affiliations:** 1 Department of Biochemistry, University of Cambridge, Tennis Court Road, Cambridge CB2 1QW, UK

## Abstract

**Background:**

CCCH type zinc finger proteins are RNA binding proteins with regulatory functions at all stages of mRNA metabolism. The best-characterized member, tritetraproline (TTP), binds to AU rich elements in 3' UTRs of unstable mRNAs, mediating their degradation. In kinetoplastids, CCCH type zinc finger proteins have been identified as being involved in the regulation of the life cycle and possibly the cell cycle. To date, no systematic listing of CCCH proteins in kinetoplastids is available.

**Results:**

We have identified the complete set of CCCH type zinc finger proteins in the available genomes of the kinetoplastid protozoa *Trypanosoma brucei*, *Trypanosoma cruzi *and *Leishmania major*. One fifths (20%) of all CCCH motifs fall into non-conventional classes and many had not been previously identified. One third of all CCCH proteins have more than one CCCH motif, suggesting multivalent RNA binding. One third have additional recognizable domains. The vast majority are unique to Kinetoplastida or to a subgroup within. Two exceptions are of interest: the putative orthologue of the mRNA nuclear export factor Mex67 and a 3'-5' exoribonuclease restricted to Leishmania species. CCCH motifs are absent from these proteins in other organisms and might be unique, novel features of the Kinetoplastida homologues. Of the others, several have a predicted, and in one case experimentally confirmed, connection to the ubiquitination pathways, for instance a HECT-type E3 ubiquitin ligase. The total number of kinetoplastid CCCH proteins is similar to the number in higher eukaryotes but lower than in yeast. A comparison of the genomic loci between the Trypanosomatidae homologues provides insight into both the evolution of the CCCH proteins as well as the CCCH motifs.

**Conclusion:**

This study provides the first systematic listing of the Kinetoplastida CCCH proteins. The number of CCCH proteins with more then one CCCH motif is larger than previously estimated, due to the identification of non-conventional CCCH motifs. Experimental approaches are now necessary to examine the functions of the many unique CCCH proteins as well as the function of the putative Mex67 and the Leishmania 3'-5' exoribonuclease.

## Background

Pathogenic kinetoplastid protozoa, such as the widely studied 'Tritryps' *Trypanosoma cruzi *(Tc), *Leishmania major *(Lm) and *Trypanosoma brucei *(Tb), have complex biphasic life cycles and consequently require changes in gene expression in response to extrinsic and intrinsic signals. For instance, at least 5% of all Tb genes are developmentally regulated at the mRNA level between any two of the experimentally tractable life cycle stages [1-4]. Kinetoplastids regulate protein coding gene expression almost exclusively at the post-transcriptional level with the aid of RNA binding proteins (reviewed in [5]). One group of RNA binding proteins is defined by the presence of a CCCH type zinc finger motif that directly binds to RNA. Different CCCH proteins regulate all stages of mRNA life, amongst the best-studied are the proteins of the TIS11 family, with the best characterized being the mammalian protein tritetraproline (TTP). TIS11 proteins bind to AU-rich elements in the 3' UTRs of their target mRNAs, in most cases mediating their degradation (reviewed in [6]). The likely mechanism is the recruitment of mRNA degradation factors to the target mRNAs, many of which have been found to interact with TIS11 proteins [7-9]. Other CCCH proteins control the translation of their target mRNAs, for instance the *C. elegans *protein POS-1 [10,11]. The Drosophila CCCH protein ZC3H3 regulates mRNA adenylation and nuclear export and also binds to known nuclear export factors [12]. Five Arabidopsis CCCH proteins have been shown to possess intrinsic endonuclease activity, including the orthologue to the polyadenylation specificity factor CPSF30 [13,14]. CCCH proteins have between 1 and 6 CCCH motifs. These were originally defined as C-X_6-14_-C-X_4-5_-C-X_3_-H [15] but recently redefined as C-X_4-15_-C-X_4-6_-C-X_3_-H, following the genome wide analysis of the rice and Arabidopsis CCCH proteins [16].

As part of a project that aimed to understand the regulation of nuclear export in trypanosomes, a putative orthologue to the yeast nuclear export factor Mex67 was identified in *T. brucei*. The finding of a CCCH motif in the putative Mex67 prompted us to set out to identify and compare the entire set of CCCH proteins in the genomes of the Kinetoplastida. A previous screen for the two most common CCCH motifs (C-X_7-_C-X_5_-C-X_3_-H and C-X_8-_C-X_5_-C-X_3_-H) in the Tritryp genomes identified of 50, 68, 41 CCCH proteins in Tb, Tc and Lm, respectively [17,18]. In addition, some proteins containing one of the common CCCH motifs also contained a C-X_10-_C-X_5_-C-X_3_-H motif [17,18]. For ease of reading here, CCCH motifs in the two most common classes C-X_7-_C-X_5_-C-X_3_-H and C-X_8-_C-X_5_-C-X_3_-H will be called 'conventional'; others, such as C-X_10-_C-X_5_-C-X_3_-H, 'non-conventional'. This term is used to highlight a difference and does not mean that they are less likely to be CCCH motifs [15].

Only three of the CCCH zinc finger proteins were identified as having readily apparent orthologues in other organisms: the splicing factor U2AF35 [19] and two components of the mRNA cleavage and polyadenylation apparatus, CPSF30 and FIP1 [20,21]. Of the previously identified CCCH proteins unique to kinetoplastids, two families have been experimentally characterized: (i) the ZFP CCCH proteins involved in the regulation of differentiation [22-25] and (ii) the cycle sequence binding proteins (CSBPs) that bind a conserved sequence in S-phase regulated mRNAs [26-28]. The vast majority of the trypanosome CCCH proteins defined by the conventional CCCH motifs appeared to have only one CCCH finger; while nearly two thirds of the Arabidopsis and rice proteins have at least two [16]. The binding of the CCCH protein TTP to AU rich elements is dependent on two intact CCCH motifs, one is not sufficient [29], and it has been speculated that in trypanosomes such multivalent RNA binding may be achieved by oligomerization, such as occurs between the CCCH proteins of the ZFP family [24,30].

Here, an extended analysis of the CCCH type zinc finger proteins in the genomes of the Tritryps is presented. The inclusion of non-conventional CCCH motifs into the search increased the fraction of CCCH proteins with more than one CCCH motif to one third and resulted in the identification of many novel CCCH proteins. One example is the putative orthologue to the nuclear export factor Mex67 that has no CCCH motifs in mammals or fungi.

## Results and Discussion

### Identification of CCCH proteins in kinetoplastids

**Figure 1:**
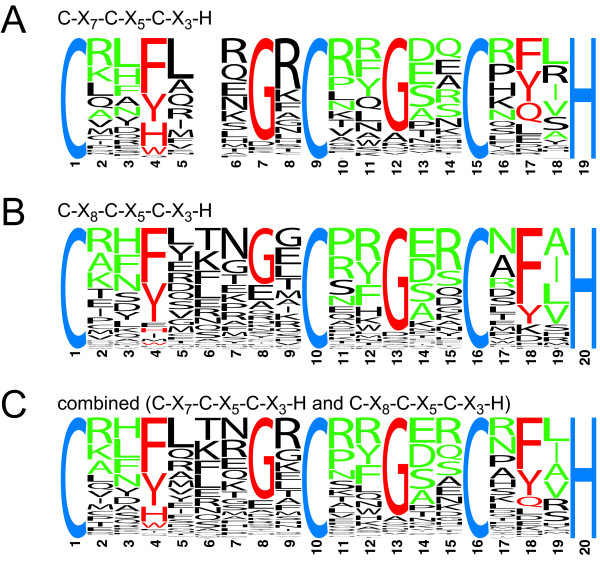
**Sequence logos**. Combined sequence logos of **A) **Tritryp C-X_7_-C-X_5_-C-X_3_-H motifs, **B) **Tritryp C-X_8_-C-X_5_-C-X_3_-H motifs and **C) **Tritryp C-X_7_-C-X_5_-C-X_3_-H and C-X_8_-C-X_5_-C-X_3_-H motifs. Only CCCH motifs that were recognized by at least either Smart, Pfam or InterPro are included. Tc CCCH motifs present more than once due to allelic variants are not included to avoid any bias towards the Tc motif.

**Figure 2:**
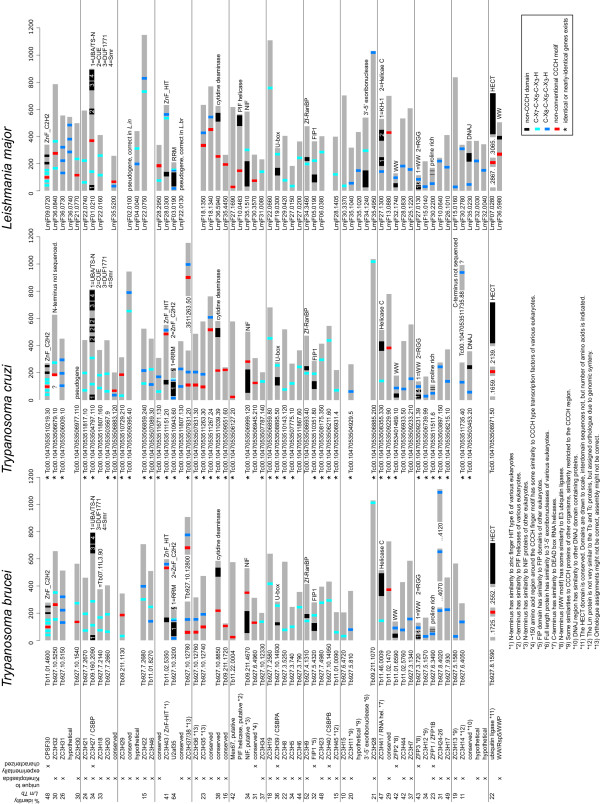
**CCCH proteins of the Tritryps**. All CCCH proteins of the final list are shown to scale. Conventional (blue) and non-conventional (red) CCCH motifs as well as any recognizable domains are indicated. The identities between the Tb and Lm homologues (first column) were calculated using the needle algorithm of Emboss pairwise alignments.

**Figure 3:**
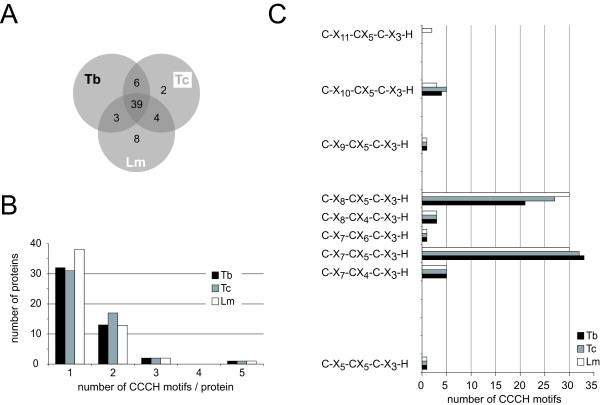
**Statistics on kinetoplastid CCCH proteins**. A) Numbers of CCCH proteins present in all Tritryps or the respective subset. **(B) **Numbers of CCCH proteins with 1, 2, 3, or 5 CCCH motifs. **C) **Numbers of CCCH motifs per CCCH-motif-class.

The Tritryp genome database [31] was searched for all proteins containing the motif C-X_4-15_-C-X_4-6_-C-X_3_-H. This motif covers both the conventional (C-X_7-_C-X_5_-C-X_3_-H and C-X_8-_C-X_5_-C-X_3_-H) and the more recently defined non-conventional CCCH motifs. Some motifs were recognized more than once due to multiple cysteine residues; the sequence closest to a conventional motif was used for further analysis. Protein sequences derived from genes annotated as pseudogenes in the databases were removed after the initial screen (see materials and methods for details). The resulting set contained 131, 233 and 120 proteins from Tb, Tc and Lm, respectively, containing between one and five putative CCCH motifs. The larger number for Tc results from pairs of allelic variants as the genome strain (CL Brener) is a hybrid [32]. The CCCH motifs belonged to all possible classes, but there was a clear enrichment in the two conventional CCCH motifs. Of all candidate CCCH motifs, there were 52/158 (Tb), 106/292 (Tc) and 47/148 (Lm) conventional CCCH motifs that were also recognized by one or more of SMART (Sm00356), Pfam (PF00642) or InterPro (IPR000571). These 205 CCCH motifs (the training set) were used to produce sequence logos [Additional file 1]. There was very little difference between the sequence logos derived from each of the Tritryps [Additional file 1] and little difference between the sequence logos of the two conventional classes of CCCH motifs (Figure 1A and 1B). As expected for CCCH motifs that are recognized by Pfam, the logo was similar to the Pfam sequence logo for CCCH motifs or the logos for rice and Arabidopsis CCCH proteins [16]. A combined sequence logo was created (Figure 1C) and used to evaluate all non-conventional CCCH motifs and conventional CCCH motifs not recognized by Pfam, Smart or InterPro. First, a CCCH motif candidate was considered a likely CCCH motif, if it had at least 2/4 of the conserved residues coloured red in the sequence logo in Figure 1: F/Y/H/W at position C1+3, G at position C2-2, G at position C3-3 or F/Y/Q at position C3+2. This criterion was stringent as it excluded 5 of the 205 sequences in the training set as well as the cycling sequence binding protein A (CSBPA) of Lm; the Tb and Tc orthologues of CSBPA were recognized. Therefore, in a second step, CCCH finger candidates not recognized by the first criterion were also considered likely CCCH motifs, if they had three or more identities to the amino acids coloured either red or green in the sequence logo in Figure 1. This way, all 205 CCCH fingers of the training set and *Lm*CSBPA were included. The list now contained 66 (Tb), 133 (Tc), and 78 (Lm) candidate CCCH zinc finger proteins. In a third step, proteins that were unlikely to be CCCH zinc finger proteins were removed manually: (i) cell surface proteins or proteins with a known function unrelated to nucleic acids and (ii) CCCH motif candidates with homologues in the other Tritryps that did not possess the CCCH motif. In most cases, the manually excluded proteins had non-conventional CCCH motifs that had little similarity to the sequence logo, supporting the finding that they were not real CCCH zinc fingers. The exceptions were five proteins that were classified as 'possible CCCH zinc finger proteins'. These were excluded from the final list but are discussed below. After removing allelic variants from the Tc set, the list of CCCH proteins contained 51 (Tb), 53 (Tc) and 54 (Lm) sequences [Additional file 2]. These numbers were reduced to 48 (Tb), 51 (Tc) and 54 (Lm) after CCCH proteins encoded by genes that were present in tandem arrays of the same genomic locus and had more than 40% amino acid sequence identity to each other (e.g. gene products of recently duplicated genes) were counted as one in order to limit redundancy. The complete list of genes is shown schematically in Figure 2. Together, the Tritryps have 62 non-redundant CCCH proteins. Of these, 39 are present in all three species; the remaining 23 are unique to one or two of the Tritryps (Figure 3A, Figure 2). Roughly one third, 16/48 (Tb), 20/51 (Tc), and 16/54 (Lm), have more than one CCCH motif (Figure 3B, Figure 2). Many of these have one non-conventional CCCH motif, the reason they had not been classified previously as containing multiple CCCH fingers. 20% of all CCCH motifs were non-conventional with C-X_7-_C-X_4_-C-X_3_-H, C-X_8-_C-X_4_-C-X_3_-H and the previously identified C-X_10-_C-X_5_-C-X_3_-H being the most abundant non-conventional CCCH motifs (Figure 3C) [Additional File 2]. About one third of all CCCH proteins, 14/48 (Tb), 15/51 (Tc) and 18/54 (Lm), have other recognizable domains (Figure 2). In addition to the domains present in the experimentally characterized CCCH proteins described above, recognizable domains included other zinc finger domains (ZnF_C2H2, Zf-RanBP, ZnF_HIT), a cytidine deaminase domain, a helicase domain, a DNAJ domain, an exonuclease domain, a HECT domain and a U-box. The vast majority of CCCH proteins are unique to Kinetoplastida, or even to a subgroup of the Kinetoplastida. Most have no similarities to proteins of other eukaryotes; some have similarities that are restricted to conserved domains only. Two exceptions were a putative Mex67 orthologue and a Leishmania specific 3' exoribonuclease, discussed below. Using the identity between Tb and Lm for illustration, the most conserved protein was the splicing factor U2af35 (64%), followed by ZC3H9 (52%), ZC3H17 (49%), CPSF30 (48%), ZC3H23 (48%) and ZC3H41 (48%) (Figure 2).

### A CCCH motif in a putative Mex67/TAP/NXF1

This investigation of CCCH proteins was initiated by an *in silico *search for a trypanosome homologue of the budding yeast mRNA export factor Mex67 (NXF1 and TAP in mammalian cells, reviewed in [33]). Using standard BLAST parameters, the protein encoded by Tb11.22.0004 gave the highest p-value (2.3e-07) and screening the *S. cerevisiae *proteome with Tb11.22.0004 gave a single hit, Mex67, p-value (1.5e-07) [Additional file 3:Supplemental Figure S2A]. An InterPro search for domains and motifs in Tb11.22.0004 detected the presence of PTHR10662 [34], characteristic of NXF1-related proteins, and a CCCH zinc finger near the N-terminus. *S. cerevisiae *Mex67 and mammalian NXF1 do not contain a zinc finger [Additional file 3:Supplemental Figure S2A].

Members of the NXF1 family are generally not very similar to each other, for example, the region of highest identity between the *Drosophila melanogaster *NXF1 and *S. cerevisiae *Mex67 (residues 107-598, determined by NCBI blast2seq) is 23% identical. Using the same programme, the region of highest identity between *S. cerevisiae *Mex67 and Tb11.22.0004 was identified as ~160 amino acids at the N-termini (Mex67 residues 96-248 and Tb11.22.0004 residues 49 to 206) and had 31% identity. The same identity was found between Drosophila NXF1 and *S. cerevisiae *Mex67 for the same region [Additional file 3:Supplemental Figure S2B]. The closest homologues of Tb11.22.004 in the other Tritryps, Tc00.1047053506127.20/Tc00.1047053508271.4 and LmjF27.1690 also contain a zinc finger near the N-terminus and have closest homology to the yeast mRNA nuclear export factor Mex67 and its mammalian orthologue TAP/NXF1.

**Figure 4:**
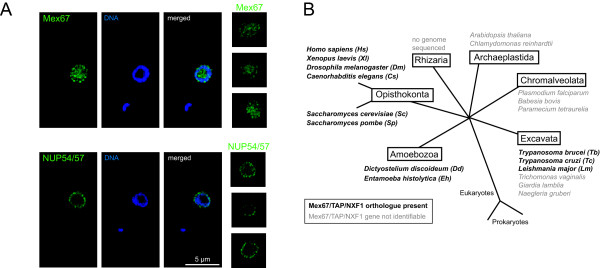
**Mex67/TAP/NXF1**. A) A C-terminally tagged eYFP fusion protein of the *T. brucei *putative Mex67 orthologue (Tb11.22.0004) was expressed from its endogenous locus in procyclic trypanosomes. Single plane confocal microscopy images are shown. For comparison, single plane confocal microscopy images of cells expressing an eYFP fusion protein of the nucleoporin NUP54/57 are shown. **B) **Available genomes of organisms that are representative for five of the six major groups of the eukaryotic kingdom were searched for proteins homologous to the *S. cerevisiae *Mex67 by Blast. Organisms that have an identifiable Mex67 gene are shown in black/bold, organisms without a readily identifiable Mex67 homologue are shown in grey.

The subcellular localisation of the putative Tb MEX67 was determined by fluorescent protein tagging of the endogenous locus of Tb11.02.0004. The protein localized to the nucleoplasm as well as to spots at the nuclear periphery that are similar in number and size to spots seen with an eYFP fusion of the nucleoporin NUP54/57 (Figure 4A). The localization of Mex67 to nuclear pores has been described for both yeast Mex67 [35] and the mammalian homologue TAP [36]. This observation supports, but does not prove, the identification of Tb11.02.0004 as the Mex67 orthologue.

The finding that the putative kinetoplastid MEX67 contains an RNA-binding zinc finger is novel and Mex67 homologues from other non-Opisthokonta species were investigated. Neither the Mex67 homologues from *Dictyostelium discoideum *nor from *Entamoeba histolytica *(both Amoebozoa) contained a CCCH domain. No close Mex67 homologue was readily recognisable in the available genome sequences of organisms in either the Archaeplastida (plants) or the Chromalveolata (Figure 4B). Furthermore, other than in kinetoplastids, Mex67 homologues were not readily recognisable in other Excavata species: *Naegleria gruberi*, *Giardia lamblia *and *Trichomonas vaginalis*. The role of the CCCH motif in the putative trypanosome MEX67 remains unknown, but it might indicate differences in the regulation of mRNA nuclear export between trypanosomes and other organisms.

### A CCCH protein with a 3' exoribonuclease domain

The Leishmania-specific 3' exoribonuclease, LmjF34.1240, is similar to 3' exoribonucleases of various eukaryotes and the region of homology is not restricted to the exoribonuclease domain but extends over the entire protein [Additional file 4]. The CCCH motif, however, is unique to the Leishmania protein. Several Arabidopsis CCCH proteins possess intrinsic nuclease activity, including CPSF30 and Smic1 [13,14]. However, neither of these proteins has a clearly defined nuclease domain and the nuclease activity was dependent on one (CPSF30) or two (Smic1) of the CCCH motifs. To our knowledge, the Leishmania protein is the first protein that has both a 3' exoribonuclease domain and a CCCH motif and it would be very interesting to examine whether the CCCH motif is involved in regulating exoribonuclease activity and/or substrate specificity. The exoribonuclease is present in all Leishmania species, but absent from all trypanosome species. It is tempting so speculate that the enzyme might be involved in mRNA regulation via the *cis*-acting element SIDER (Short Interspersed DEgenerated Retrotransposon). SIDER elements are mainly found in 3' UTRs of Leishmania genes, where they promote mRNA degradation (SIDER2, [37]) or regulate translation [38,39]. In contrast, SIDER elements are 70 times less abundant in Tb and usually found in the subtelomeric regions [37] and do not appear to function as regulatory *cis*-acting elements of mRNAs.

### CCCH finger proteins and ubiquitination

Ubiquitination requires the ubiquitin-activating enzyme (E1), the ubiquitin conjugating enzyme (E2) and the ubiquitin protein ligase (E3). Substrate specificity is usually determined by the E3 ligase. Three different types of E3 ligases can be distinguished, named after their catalytic domains: the RING type, the (RING-related) U-box type and the HECT type. Substrates can either be monoubiquitinated, multi-ubiquitinated (monoubiquitinated on multiple lysine residues) or polyubiquitinated (carrying a chain of ubiquitins). Depending on the type and place of ubiquitination, ubiquitinated proteins can be either targeted for degradation by the 26S proteosome or the ubiquitination acts as a signal, for example to change the intracellular localization of the protein.

Among the kinetoplastid CCCH proteins are several with a predicted, and in one case experimentally confirmed, connection to ubiquitination. Both ZFP2 and ZFP3 have a motif upstream of the WW domain that has closest homology to a motif upstream of the WW domain of HECT type E3 ligases of the Nedd4 family [22,24] (Figure 2). CSBP (ZC3H27) has two different types of ubiquitin interacting domains: UBA (ubiquitin associated domain) and CUE [28] (Figure 2). UBA domains have highest affinity for polyubiquitin; CUE domains, in contrast, have been shown to bind to monoubiquitin and promote autoubiquitination (reviewed in [40]). In fact, a fraction of the Leishmania CSBP protein has been shown to be monoubiquitinated and a small fraction is either polyubiquitinated or multi-monoubiquitinated [28], consistent with the CUE domain mediating autoubiquitination. Two putative E3 ubiquitin ligases are among the Kinetoplastida CCCH finger proteins: one U-box type and one HECT-type E3 ubiquitin ligase (Figure 2).

Proteins with a combination of domains associated with ubiquitination and RNA binding are common in many species [41]; trypanosomes are no exception. Ubiquitination may play important roles in the regulation of stability or localization of RNA binding proteins. *C. elegans *oogenesis and embryogenesis provide two good examples: five germline specific CCCH proteins (PIE-1, POS-1, MEX-1, MEX-5, MEX06) are degraded in somatic cells via binding of the CCCH-finger binding protein ZIF-1 to the CCCH motif and recruitment of an E3 ubiquitin ligase [42]. Oma-1, a CCCH protein involved in asymmetric distribution of determinants in the egg is degraded during the first zygotic cell cycle via a ZIF-1-independent E3 ubiquitin ligase complex [43].

Four of the trypanosome CCCH proteins that have a predicted or known connection to ubiquitination are involved in the regulation of the life cycle or cell cycle. In one case, CSBP, ubiquitination of the CCCH protein has been shown. It is possible that ubiquitination is a mechanism to quickly change either stability or intracellular localization of CCCH proteins in response to life and/or cell cycle triggers.

### Origin of the kinetoplastid CCCH proteins

Most CCCH proteins are present in all three Tritryps (Figure 3A); the average amino acid sequence identity between the closest homologues in Tb and Lm is 32% (Figure 2). Nevertheless, there are variations in the CCCH protein content between the Tritryps (Figure 2) and to examine the origin of these differences, the synteny between the genomic loci coding for the Tb and the Lm CCCH proteins was examined.

**Figure 5:**
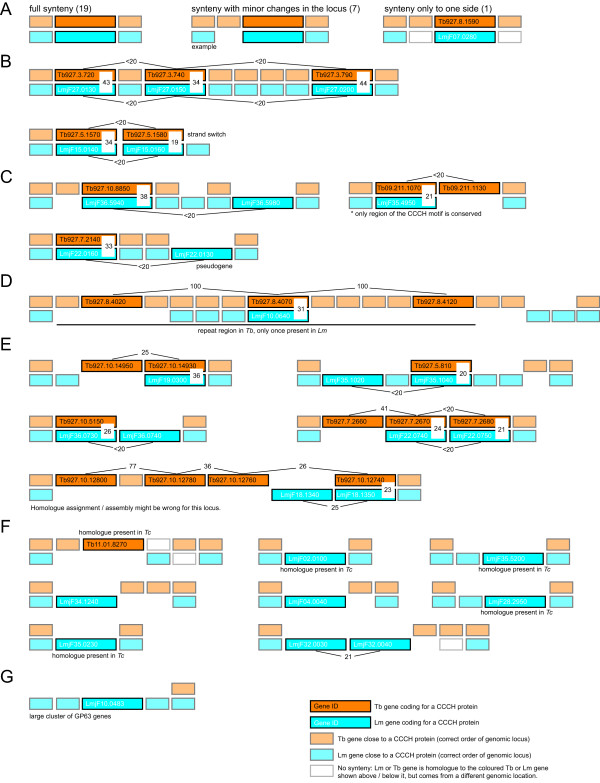
**Comparison between the genomic loci of the Lm and Tb CCCH proteins**. The genomic loci of all CCCH proteins of Tb and Lm are shown as schematic drawings, each bar representing one gene. Filled bars represent full synteny while open bars represent orthologous genes that come from a different genomic location (either from a completely different chromosome or from the same genomic locus but at a different position). Numbers present the percentage amino acid identity between the gene products of the homologous genes or the duplicated genes as determined by Emboss pairwise alignments (gap penalty:10.0; extend penalty: 0.5; matrix: EBLOSUM62). The values for global identity are shown.

The CCCH proteins of Tb and Lm are distributed over 47 genomic loci (Figure 5). 27 of these loci contain single CCCH proteins; of these, 19 have full synteny, seven have synteny with minor changes close to the locus and one has synteny to one side only (Figure 5A). Another 11 loci contain tandemly repeated genes in Tb and/or Lm, with at least one pair between the species (Figure 5B-E). These 11 fall into four different groups: two loci have full synteny with significantly higher similarity between the homologous pairs than between the duplicated genes within the locus, indicating that gene duplication took place before the separation of Tb and Lm (Figure 5B). Three loci have a duplicated gene present in only one of the species, but with significantly higher similarity between the homologues from the two species than between the duplicated genes, probably best explained by loss of one of the duplicated genes from one species after the separation of Tb and Lm (Figure 5C). One locus has three identical copies of a Tb gene, while the syntenic Lm locus has only one gene with 31% identity to the Tb genes, indicating that the gene duplication probably occurred after divergence (Figure 5D). The remaining five loci also contain differences between the two species in the number of duplicated genes, but it remains unclear, whether these originated from a gene duplication event in one of the species or from the loss of a gene in the other (Figure 5E).

Nine loci have a CCCH protein gene in only one of the two species (Figure 5F and 5G). For eight loci, regional synteny is still intact, usually with one or two other genes out of synteny in addition to the gene coding for the CCCH proteins (Figure 5F). In one case, synteny was lost (Figure 5G). Are the differences between these loci due to the loss or the gain of the gene in one of the species? Four of the Lm genes are also present in Tc, indicating that the loss of the Tb gene is the more likely scenario, since Leishmanias separated from Trypanosomes before the separation of Tb and Tc. Four of the Lm genes are absent from both Tc and Tb, suggesting that they either arose after the separation of Leishmania from the trypanosomes or were lost after the separation of Leishmania in the common ancestor of Tc and Tb. One gene is present in Tb and Tc, but not in Lm, indicating that it was either lost in Leishmania, or gained in the common ancestor of Tc and Tb.

Taken together, the analysis confirms that the majority of the kinetoplastid CCCH proteins genes evolved prior to the separation of the species and the presence of many loci with more than one CCCH protein gene suggests that gene duplication was important in the evolution of CCCH proteins. Differences in the content of the genes coding for CCCH proteins between Tb and Lm can be accounted for by (i) the loss or gain of a single gene or (ii) gene duplication or loss of a previously duplicated gene; in both cases this occurred with only small changes in synteny.

### Possible CCCH proteins

**Figure 6:**
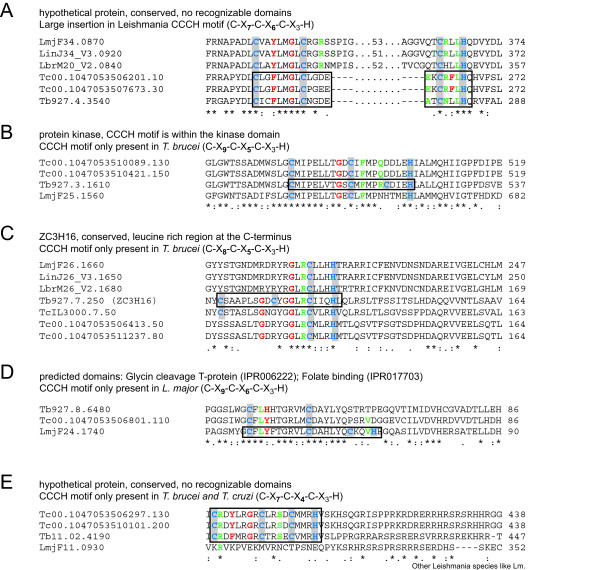
**Possible CCCH proteins**. Alignments of the homologous genomic loci of five proteins that we classified as "possible CCCH proteins" are shown. Possible CCCH proteins have conventional or close to conventional CCCH motifs that are absent from one or both of the other Tritryps. All five proteins were excluded from the final list. Amino acids are coloured as in the sequence logo in Figure 1, the cysteine and histidine residues of the CCCH motif are highlighted and intact CCCH finger motifs are framed in black.

Five proteins were classified as "possible CCCH proteins" and excluded from the final list, because the CCCH motif was absent from one or both of the other Tritryps. Alignments are shown in Figure 6 and give some insight into how CCCH motifs might have evolved. One Leishmania protein (LmjF34.0870) has a large insertion in the middle of its CCCH motif, while the CCCH motifs of Tb and Tc are intact (Figure 6A). It is unlikely that the Leishmania protein contains a zinc finger. A Tb protein (Tb927.3.1610) has a CCCH motif in the middle of a kinase domain; the motif is absent from the other Tritryps despite of the high degree of conversion in this region (Figure 6B). Similarly, the previously classified Tb zinc finger protein ZC3H16 (Tb927.7.250) has a conventional CCCH motif that is absent from the closest homologues in Tc and Lm (Figure 6C). In this case, the selective loss or gain of a CCCH motif in evolution is clearly visible: *T. congolense*, a close relative to Tb, has two cysteine residues of the CCCH motif, while Lm and Tc have only one. A similar case for a Leishmania protein (LmjF24.1740) is shown in Figure 6D. In another case, a C-X_7_-C-X_4_-C-X_3_-H motif is present in Tb and Tc (Tb11.02.4190; Tc00.1047053506297.130; Tc00.1047053510101.200), but absent from Leishmania (LmjF11.0930). However, the Leishmania protein has very little homology to its Tb and Tc homologues in the region of the CCCH motif (Figure 6E). In this case it is likely that the CCCH motif was lost or gained by larger genomic rearrangements and it might be fully functional in Tb and Tc. Without experimental data it is impossible to predict whether either of the possible proteins CCCH proteins have functional CCCH type zinc fingers.

### Comparison with other eukaryotes

The number of non-redundant CCCH proteins in the Tritryps (48 in Tb, 51 in Tc, 54 in Lm) is similar to the number in higher eukaryotes: Arabidopsis, rice, mouse and human have 68, 67, 58 and 55 predicted CCCH proteins, respectively [16,44]. The fraction of CCCH proteins with more than one CCCH motif (34%) is about half of the fraction found among the rice (64.2%) or Arabidopsis (63.2%) CCCH proteins.

It has been previously suggested that kinetoplastids have significantly more CCCH proteins than other lower eukaryotes, based on a comparison with yeast. In the absence of published data, we attempted an estimation by screening protozoa and yeast genomes for the two conventional CCCH motifs and, as a background control, for two non-existing ones (C-X_7_-C-X_7_-C-X_3_-H and C-X_8_-C-X_7_-C-X_3_-H), without any filtering or evaluation steps. The results are summarized in Table 1: kinetoplastids have slightly more CCCH motifs than any other lower eukaryotes investigated, but they are closely followed by *T. gondii *and *D. discoideum*. Other protozoa and yeast do have fewer CCCH motifs. These data are an estimation, but they do suggest that the number of CCCH proteins in trypanosomes is only slightly higher than in some protozoa, while other protozoa and the two yeasts have fewer CCCH proteins.

**Table 1:** Estimation of the numbers of conventional CCCH motifs in protozoa and yeast.

Organism	No of CCCH motifs (unfiltered) Y/Z (C-X_Y_-C-X_Z_-C-X_3_-H)				Genome			Total number of CCCH motifs (background corrected)
	**7/5**	**8/5**	**7/7**	**8/7**	**Size (Mbp)**	**No of genes**	**Ref**.	

*T. brucei*	37	28	4	2	26.1	9068	[59]	59
*L. major*	31	31	5	6	32.8	8272	[17]	51
*T. cruzi*	62	59	3	8	60.4	22570	[60]	110
*T. annulata*	7	6	0	1	8.4	3792	[61]	12
*D. discoideum*	18	18	4	2	33.9	12500	[62]	30
*P. falciparum*	24	9	2	5	22.9	5268	[63]	26
*T. gondii GT1*	36	19	5	1	63.0	8155	[64]; ToxoDB	49
*S. pombe*	12	9	2	2	12.5	5027	[65]	17
*S. cerevisiae*	7	6	1	0	12.1	5885	[66]	12

The genomes of the organisms listed were searched for the two conventional CCCH motifs and in addition for two fantasy CCCH motifs (C-X_7_-C-X_7_-C-X_3_-H and C-X_8_-C-X_7_-C-X_3_-H). No filtering steps were performed. The total number of conventional CCCH motifs was estimated by adding the numbers of the two conventional CCCH motifs and subtracting the numbers of the two fantasy CCCH motifs.

## Conclusions

This study has identified the entire set of CCCH proteins in the available genomes of the Tritryps; there are 48 in Tb, 51 in Tc and 54 in Lm, excluding redundancy. The main findings are: (i) The fraction of CCCH proteins with more than one CCCH motif is larger than previously assumed; many of these proteins have one non-conventional CCCH motif. (ii) The putative Mex67 orthologue as well as a Leishmania-specific 3'exoribonuclease both have a CCCH motif that is not found in their counterparts in other eukaryotes. Many of the CCCH proteins have a predicted, or in one case experimentally confirmed, connection to ubiquitination pathways. (iii) Kinetoplastids do have only slightly more CCCH proteins than some other protozoa, although the number of CCCH proteins is higher than in yeast. (iv) The vast majority of the CCCH proteins are unique to kinetoplastids or to a subgroup within. The majority evolved before the separation of the Tritryps; gene duplication played an important role. Differences in the CCCH protein content between the Tritryps is mainly due to either the loss or gain of a single gene or gene duplication or loss of a previously duplicated gene; in all cases with little disruption of synteny.

The identification of CCCH proteins in this study relies entirely on *in silico *data. Some of the identified proteins might not be true CCCH zinc finger proteins, whilst others might have been missed. For instance, some putative CCCH proteins were excluded from the final list because of the absence of the CCCH motif in one or both of their closest homologue in one or both of the other Tritryps; they might, however, be true CCCH proteins. Although the majority of CCCH motifs bind RNA, a few examples of DNA binding CCCH motifs have been reported (for example [45-47]); thus, it is possible that some of the identified trypanosome CCCH proteins are not RNA-binding proteins.

Experimental approaches are now needed to verify the *in silico *data and to examine the function of the many uncharacterized proteins. Of particular interest is the function of a CCCH motif in the putative nuclear export factor Mex67 and in the Leishmania-specific 3'-5' exoribonuclease. Both CCCH motifs are unique features of the Kinetoplastida proteins and might reveal differences to other eukaryotes in mRNA metabolism.

## Methods

### Software

Sequence logos were produced using the software of [48]. All analyses of the Tritryp genomes were performed using the tools at either the Tritryps genome database [31], GeneDB http://www.genedb.org/Homepage or EBI. Sequence alignments of multiple sequences were done using ClustalW2 with default settings as provided by the server ([49]; http://www.ebi.ac.uk/Tools/clustalw2/index.html). Pairwise alignments were performed using the EMBOSS Needle programme http://www.ebi.ac.uk/Tools/emboss/align/index.html[50] for global alignments and the Water programme [51] or BLAST2seq for local alignments. Identification of protein domains was by Pfam [52], SMART [53,54], InterPro [55] or Prosite [56].

### Identification of CCCH proteins

The Tritryp genome databases [31] were searched for CCCH motif containing proteins using motif search for C-X_4-15_-C-X_4-6_-C-X_3_-H. A sequence logo was created including only C-X_7/8_-C-X_5_-C-X_3_-H motifs that were recognized by at least either SMART (Sm00356), Pfam (PF00642) or InterPro (IPR000571) (the training set) and are thus very likely to be real CCCH motifs. This consensus motif was then used to arbitrarily define conditions to further filter all CCCH motifs that did not fall into the group of the training set. The stringency of the chosen conditions was tested on the training set and gradually decreased until it included all proteins in the training set. The dataset was further filtered manually to exclude proteins unlikely to contain CCCH motifs using the criteria described in the results section.

### Identification of pseudogenes

The dataset contained four genes that were annoted as pseudogenes in the genome databases, two in *L. major *(LmjF02.0100 and LmjF22.0130) and two in *T. cruzi *(Tc00.1047053506977.110 and Tc00.1047053511715.50). Both Leishmania pseudogenes have premature stop codons and longer counterparts in other Leishmania strains (see Figure 2). Confirmation of the sequence for the *Leishmania major *isoforms was obtained from Matt Rogers (Sanger institute). Tc00.1047053506977.110 also has a premature stop codon and a longer counterpart in *T. congolense*. Tc00.1047053511715.50 has an internal shift out of frame and then back in again, the CCCH motif is in the out of frame region and therefore an artefact.

### Intracellular localization of Mex67

The putative *T. brucei *Mex67 orthologue (Tb11.22.0004) and NUP54/57 (Tb927.4.5200) were expressed in *Trypanosoma brucei *Lister 427 procyclic cells as C-terminally tagged eYFP fusion proteins from their endogenous loci as described in [57]. For microscopic imaging, cells were washed once in SDM79 without serum or haem, fixed at a density of 1*10^7 ^cells/ml with 2.4% paraformaldehyde overnight, washed once in PBS and stained with Hoechst H33258. Confocal images were prepared using a BioRad Radiance 2100 on a Nikon Eclipse E800 upright microscope using a 100/1.4 Oil DIC objective. Transgenic trypanosomes were generated using standard procedures [58].

## List of Abbreviations

Tb: *Trypanosoma brucei*; Lm: *Leishmania major*; Tc: *Trypanosoma cruzi; *Lb: *Leishmania braziliensis*; Li: *Leishmania infantum; *Tc:*Trypanosoma congolense; *CSBP: cycling sequence binding protein.

## Authors' contributions

All experimental and *in silico *work was done by SK. The manuscript was prepared by SK and MC. NCK cloned and characterized the *T. brucei *Mex67 protein. All authors approved to the content of the manuscript.

## Supplementary Material

Additional file 1 **Figure S1: equence logos of the C-X_7_-C-X_5_-C-X_3_-H and C-X_8_-C-X_5_-C-X_3_-H zinc fingers of Tb, Tc and Lm**. Only CCCH motifs that were recognized by at least either Smart, Pfam or InterPro are included. Tc CCCH motifs present more than once due to allelic variants were not included. The sequence logos shown here were used to create the combined Tritryp sequence logos shown in Figure 1. Click here for file

Additional file 2 **Table S1: List of the Tritryp CCCH proteins**. List of the Tritryp CCCH proteins, including molecular weight, class of the CCCH motif and redundant genes. Click here for file

Additional file 3 **Figure S2: Alignment of the putative TbMex67 with homologues of other organisms**. A) Alignment of *S. cerevisiae *Mex67 with *T. brucei *Tb11.22.0004 B) Alignment of region of greatest identity between *S. cerevisiae *Mex67, Tb11.22.0004 and *D. melanogaster *NXF1. Click here for file

Additional file 4 Figure S3: Alignment of the Leishmania 3'-5' exoribonuclease (LmjF34.1240) with the homologous proteins of *Mus musculus*. Click here for file

## References

[B1] Kabani S Fenn K Ross A Ivens A Smith TK Ghazal P Matthews K Genome-wide expression profiling of in vivo-derived bloodstream parasite stages and dynamic analysis of mRNA alterations during synchronous differentiation in Trypanosoma brucei BMC Genomics 2009 10 427 10.1186/1471-2164-10-427 19747379 PMC2753553

[B2] Jensen BC Sivam D Kifer CT Myler PJ Parsons M Widespread variation in transcript abundance within and across developmental stages of Trypanosoma brucei BMC Genomics 2009 10 482 10.1186/1471-2164-10-482 19840382 PMC2771046

[B3] Queiroz R Benz C Fellenberg K Hoheisel JD Clayton C Transcriptome analysis of differentiating trypanosomes reveals the existence of multiple post-transcriptional regulons BMC Genomics 2009 10 495 10.1186/1471-2164-10-495 19857263 PMC2772864

[B4] Brems S Guilbride DL Gundlesdodjir-Planck D Busold C Luu V-D Schanne M Hoheisel J Clayton C The transcriptomes of Trypanosoma brucei Lister 427 and TREU927 bloodstream and procyclic trypomastigotes Mol Biochem Parasitol 2005 139 2 163 172 10.1016/j.molbiopara.2004.11.004 15664651

[B5] Clayton C Shapira M Post-transcriptional regulation of gene expression in trypanosomes and leishmanias Mol Biochem Parasitol 2007 156 2 93 101 10.1016/j.molbiopara.2007.07.007 17765983

[B6] Baou M Jewell A Murphy JJ TIS11 family proteins and their roles in posttranscriptional gene regulation J Biomed Biotech 2009 2009 634520 10.1155/2009/634520 PMC2722025 19672455

[B7] Lykke-Andersen J Wagner E Recruitment and activation of mRNA decay enzymes by two ARE-mediated decay activation domains in the proteins TTP and BRF-1 Genes Dev 2005 19 3 351 361 10.1101/gad.1282305 15687258 PMC546513

[B8] Fenger-Gron M Fillman C Norrild B Lykke-Andersen J Multiple processing body factors and the ARE binding protein TTP activate mRNA decapping Mol Cell 2005 20 6 905 915 10.1016/j.molcel.2005.10.031 16364915

[B9] Chen CY Gherzi R Ong SE Chan EL Raijmakers R Pruijn GJ Stoecklin G Moroni C Mann M Karin M AU binding proteins recruit the exosome to degrade ARE-containing mRNAs Cell 2001 107 4 451 464 10.1016/S0092-8674(01)00578-5 11719186

[B10] Ogura K Kishimoto N Mitani S Gengyo-Ando K Kohara Y Translational control of maternal glp-1 mRNA by POS-1 and its interacting protein SPN-4 in Caenorhabditis elegans Development 2003 130 11 2495 2503 10.1242/dev.00469 12702662

[B11] Tabara H Hill RJ Mello CC Priess JR Kohara Y pos-1 encodes a cytoplasmic zinc-finger protein essential for germline specification in C. elegans Development 1999 126 1 1 11 9834181 10.1242/dev.126.1.1

[B12] Hurt JA Obar RA Zhai B Farny NG Gygi SP Silver PA A conserved CCCH-type zinc finger protein regulates mRNA nuclear adenylation and export J Cell Biol 2009 185 2 265 277 10.1083/jcb.200811072 19364924 PMC2700372

[B13] Addepalli B Hunt AG A novel endonuclease activity associated with the Arabidopsis ortholog of the 30-kDa subunit of cleavage and polyadenylation specificity factor Nucleic Acids Res 2007 35 13 4453 4463 10.1093/nar/gkm457 17576667 PMC1935010

[B14] Addepalli B Hunt AG Ribonuclease activity is a common property of Arabidopsis CCCH-containing zinc-finger proteins FEBS Lett 2008 582 17 2577 2582 10.1016/j.febslet.2008.06.029 18582464

[B15] Berg JM Shi Y The galvanization of biology: a growing appreciation for the roles of zinc Science 1996 271 5252 1081 1085 10.1126/science.271.5252.1081 8599083

[B16] Wang D Guo Y Wu C Yang G Li Y Zheng C Genome-wide analysis of CCCH zinc finger family in Arabidopsis and rice BMC Genomics 2008 9 44 10.1186/1471-2164-9-44 18221561 PMC2267713

[B17] Ivens AC Peacock CS Worthey EA Murphy L Aggarwal G Berriman M Sisk E Rajandream MA Adlem E Aert R The genome of the kinetoplastid parasite, Leishmania major Science 2005 309 5733 436 442 10.1126/science.1112680 16020728 PMC1470643

[B18] Hendriks E Matthews K Post-Transcriptional Control of Gene Expression in African Trypanosomes Trypanosomes After the Genome 2007 209 237

[B19] Vazquez M Atorrasagasti C Bercovich N Volcovich R Levin MJ Unique features of the Trypanosoma cruzi U2AF35 splicing factor Mol Biochem Parasitol 2003 128 1 77 81 10.1016/S0166-6851(03)00007-0 12706799

[B20] Hendriks EF Abdul-Razak A Matthews KR tbCPSF30 depletion by RNA interference disrupts polycistronic RNA processing in Trypanosoma brucei J Biol Chem 2003 278 29 26870 26878 10.1074/jbc.M302405200 12746436

[B21] Bercovich N Levin MJ Vazquez MP The FIP-1 like polyadenylation factor in trypanosomes and the structural basis for its interaction with CPSF30 Biochem Biophys Res Commun 2009 380 4 850 855 10.1016/j.bbrc.2009.01.182 19338765

[B22] Hendriks EF Robinson DR Hinkins M Matthews KR A novel CCCH protein which modulates differentiation of Trypanosoma brucei to its procyclic form Embo J 2001 20 23 6700 6711 10.1093/emboj/20.23.6700 11726506 PMC125759

[B23] Hendriks EF Matthews KR Disruption of the developmental programme of Trypanosoma brucei by genetic ablation of TbZFP1, a differentiation-enriched CCCH protein Mol Microbiol 2005 57 3 706 716 10.1111/j.1365-2958.2005.04679.x 16045615 PMC2686838

[B24] Paterou A Walrad P Craddy P Fenn K Matthews K Identification and stage-specific association with the translational apparatus of TbZFP3, a CCCH protein that promotes trypanosome life-cycle development J Biol Chem 2006 281 51 39002 39013 10.1074/jbc.M604280200 17043361 PMC2688685

[B25] Walrad P Paterou A Acosta-Serrano A Matthews KR Differential trypanosome surface coat regulation by a CCCH protein that co-associates with procyclin mRNA cis-elements PLoS Pathog 2009 5 2 e1000317 10.1371/journal.ppat.1000317 19247446 PMC2642730

[B26] Mahmood R Hines JC Ray DS Identification of cis and trans elements involved in the cell cycle regulation of multiple genes in Crithidia fasciculata Mol Cell Biol 1999 19 9 6174 6182 10454564 10.1128/mcb.19.9.6174 PMC84551

[B27] Mahmood R Mittra B Hines JC Ray DS Characterization of the Crithidia fasciculata mRNA cycling sequence binding proteins Mol Cell Biol 2001 21 14 4453 4459 10.1128/MCB.21.14.4453-4459.2001 11416125 PMC87105

[B28] Bhandari D Saha P mRNA cycling sequence binding protein from Leishmania donovani (LdCSBP) is covalently modified by ubiquitination FEMS Microbiol Lett 2007 273 2 206 213 10.1111/j.1574-6968.2007.00789.x 17559572

[B29] Lai WS Carballo E Strum JR Kennington EA Phillips RS Blackshear PJ Evidence that tristetraprolin binds to AU-rich elements and promotes the deadenylation and destabilization of tumor necrosis factor alpha mRNA Mol Cell Biol 1999 19 6 4311 4323 10330172 10.1128/mcb.19.6.4311 PMC104391

[B30] Caro F Bercovich N Atorrasagasti C Levin MJ Vázquez MP Protein interactions within the TcZFP zinc finger family members of Trypanosoma cruzi: implications for their functions Biochemical and Biophysical Research Communications 2005 333 3 1017 1025 10.1016/j.bbrc.2005.06.007 15964555

[B31] Aslett M Aurrecoechea C Berriman M Brestelli J Brunk BP Carrington M Depledge DP Fischer S Gajria B Gao X TriTrypDB: a functional genomic resource for the Trypanosomatidae Nucleic Acids Res 2010 38 Database D457 462 10.1093/nar/gkp851 19843604 PMC2808979

[B32] Machado CA Ayala FJ Nucleotide sequences provide evidence of genetic exchange among distantly related lineages of Trypanosoma cruzi Proc Natl Acad Sci USA 2001 98 13 7396 7401 10.1073/pnas.121187198 11416213 PMC34680

[B33] Carmody SR Wente SR mRNA nuclear export at a glance J Cell Sci 2009 122 Pt 12 1933 1937 10.1242/jcs.041236 19494120 PMC2723150

[B34] Thomas PD Campbell MJ Kejariwal A Mi H Karlak B Daverman R Diemer K Muruganujan A Narechania A PANTHER: a library of protein families and subfamilies indexed by function Genome Res 2003 13 9 2129 2141 10.1101/gr.772403 12952881 PMC403709

[B35] Segref A Sharma K Doye V Hellwig A Huber J Luhrmann R Hurt E Mex67p, a novel factor for nuclear mRNA export, binds to both poly(A)+ RNA and nuclear pores Embo J 1997 16 11 3256 3271 10.1093/emboj/16.11.3256 9214641 PMC1169942

[B36] Katahira J Strasser K Podtelejnikov A Mann M Jung JU Hurt E The Mex67p-mediated nuclear mRNA export pathway is conserved from yeast to human Embo J 1999 18 9 2593 2609 10.1093/emboj/18.9.2593 10228171 PMC1171339

[B37] Bringaud F Müller M Cerqueira GC Smith M Rochette A El-Sayed NMA Papadopoulou B Ghedin E Members of a large retroposon family are determinants of post-transcriptional gene expression in Leishmania PLoS Pathog 2007 3 9 1291 1307 10.1371/journal.ppat.0030136 17907803 PMC2323293

[B38] Boucher N Wu Y Dumas C Dube M Sereno D Breton M Papadopoulou B A common mechanism of stage-regulated gene expression in Leishmania mediated by a conserved 3'-untranslated region element J Biol Chem 2002 277 22 19511 19520 10.1074/jbc.M200500200 11912202

[B39] McNicoll F Müller M Cloutier S Boilard N Rochette A Dubé M Papadopoulou B Distinct 3'-untranslated region elements regulate stage-specific mRNA accumulation and translation in Leishmania J Biol Chem 2005 280 42 35238 35246 10.1074/jbc.M507511200 16115874

[B40] Hurley JH Lee S Prag G Ubiquitin-binding domains Biochem J 2006 399 3 361 372 10.1042/BJ20061138 17034365 PMC1615911

[B41] Anantharaman V Koonin EV Aravind L Comparative genomics and evolution of proteins involved in RNA metabolism Nucleic Acids Res 2002 30 7 1427 1464 10.1093/nar/30.7.1427 11917006 PMC101826

[B42] DeRenzo C Reese KJ Seydoux G Exclusion of germ plasm proteins from somatic lineages by cullin-dependent degradation Nature 2003 424 6949 685 689 10.1038/nature01887 12894212 PMC1892537

[B43] Shirayama M Soto MC Ishidate T Kim S Nakamura K Bei Y Heuvel S van den Mello CC The Conserved Kinases CDK-1, GSK-3, KIN-19, and MBK-2 Promote OMA-1 Destruction to Regulate the Oocyte-to-Embryo Transition in C. elegans Curr Biol 2006 16 1 47 55 10.1016/j.cub.2005.11.070 16343905

[B44] Liang J Song W Tromp G Kolattukudy PE Fu M Genome-wide survey and expression profiling of CCCH-zinc finger family reveals a functional module in macrophage activation PLoS One 2008 3 8 e2880 10.1371/journal.pone.0002880 18682727 PMC2478707

[B45] Hwang ES Choi A Ho IC Transcriptional regulation of GATA-3 by an intronic regulatory region and fetal liver zinc finger protein 1 J Immunol 2002 169 1 248 253 12077251 10.4049/jimmunol.169.1.248

[B46] Wang L Xu Y Zhang C Ma Q Joo SH Kim SK Xu Z Chong K OsLIC, a Novel CCCH-Type Zinc Finger Protein with Transcription Activation, Mediates Rice Architecture via Brassinosteroids Signaling PLoS One 2008 3 10 e3521 10.1371/journal.pone.0003521 18953406 PMC2567845

[B47] Pomeranz MC Hah C Lin PC Kang SG Finer JJ Blackshear PJ Jang JC The Arabidopsis tandem zinc finger protein AtTZF1 traffics between the nucleus and cytoplasmic foci and binds both DNA and RNA Plant Physiol 152 1 151 165 10.1104/pp.109.145656 19897605 PMC2799353

[B48] Crooks GE Hon G Chandonia JM Brenner SE WebLogo: a sequence logo generator Genome Res 2004 14 6 1188 1190 10.1101/gr.849004 15173120 PMC419797

[B49] Larkin MA Blackshields G Brown NP Chenna R McGettigan PA McWilliam H Valentin F Wallace IM Wilm A Lopez R Clustal W and Clustal X version 2.0 Bioinformatics 2007 23 21 2947 2948 10.1093/bioinformatics/btm404 17846036

[B50] Needleman SB Wunsch CD A general method applicable to the search for similarities in the amino acid sequence of two proteins J Mol Biol 1970 48 3 443 453 10.1016/0022-2836(70)90057-4 5420325

[B51] Smith TF Waterman MS Identification of common molecular subsequences J Mol Biol 1981 147 1 195 197 10.1016/0022-2836(81)90087-5 7265238

[B52] Finn RD Tate J Mistry J Coggill PC Sammut SJ Hotz HR Ceric G Forslund K Eddy SR Sonnhammer EL The Pfam protein families database Nucleic Acids Res 2008 36 Database D281 288 18039703 10.1093/nar/gkm960 PMC2238907

[B53] Schultz J Milpetz F Bork P Ponting CP SMART, a simple modular architecture research tool: identification of signaling domains Proc Natl Acad Sci USA 1998 95 11 5857 5864 10.1073/pnas.95.11.5857 9600884 PMC34487

[B54] Letunic I Doerks T Bork P SMART 6: recent updates and new developments Nucleic Acids Res 2009 37 Database D229 232 10.1093/nar/gkn808 18978020 PMC2686533

[B55] Hunter S Apweiler R Attwood TK Bairoch A Bateman A Binns D Bork P Das U Daugherty L Duquenne L InterPro: the integrative protein signature database Nucleic Acids Res 2009 37 Database D211 215 10.1093/nar/gkn785 18940856 PMC2686546

[B56] Hulo N Bairoch A Bulliard V Cerutti L Cuche BA de Castro E Lachaize C Langendijk-Genevaux PS Sigrist CJ The 20 years of PROSITE Nucleic Acids Res 2008 36 Database D245 249 18003654 10.1093/nar/gkm977 PMC2238851

[B57] Kelly S Reed J Kramer S Ellis L Webb H Sunter J Salje J Marinsek N Gull K Wickstead B Functional genomics in Trypanosoma brucei: a collection of vectors for the expression of tagged proteins from endogenous and ectopic gene loci Mol Biochem Parasitol 2007 154 1 103 109 10.1016/j.molbiopara.2007.03.012 17512617 PMC2705915

[B58] McCulloch R Vassella E Burton P Boshart M Barry JD Transformation of monomorphic and pleomorphic Trypanosoma brucei Methods Mol Biol 2004 262 53 86 14769956 10.1385/1-59259-761-0:053

[B59] Berriman M Ghedin E Hertz-Fowler C Blandin G Renauld H Bartholomeu DC Lennard NJ Caler E Hamlin NE Haas B The genome of the African trypanosome Trypanosoma brucei Science 2005 309 5733 416 422 10.1126/science.1112642 16020726

[B60] El-Sayed NM Myler PJ Bartholomeu DC Nilsson D Aggarwal G Tran AN Ghedin E Worthey EA Delcher AL Blandin G The genome sequence of Trypanosoma cruzi, etiologic agent of Chagas disease Science 2005 309 5733 409 415 10.1126/science.1112631 16020725

[B61] Pain A Renauld H Berriman M Murphy L Yeats CA Weir W Kerhornou A Aslett M Bishop R Bouchier C Genome of the host-cell transforming parasite Theileria annulata compared with T. parva Science 2005 309 5731 131 133 10.1126/science.1110418 15994557

[B62] Eichinger L Pachebat JA Glockner G Rajandream MA Sucgang R Berriman M Song J Olsen R Szafranski K Xu Q The genome of the social amoeba Dictyostelium discoideum Nature 2005 435 7038 43 57 10.1038/nature03481 15875012 PMC1352341

[B63] Gardner MJ Hall N Fung E White O Berriman M Hyman RW Carlton JM Pain A Nelson KE Bowman S Genome sequence of the human malaria parasite Plasmodium falciparum Nature 2002 419 6906 498 511 10.1038/nature01097 12368864 PMC3836256

[B64] Gajria B Bahl A Brestelli J Dommer J Fischer S Gao X Heiges M Iodice J Kissinger JC Mackey AJ ToxoDB: an integrated Toxoplasma gondii database resource Nucleic Acids Res 2008 36 Database D553 556 18003657 10.1093/nar/gkm981 PMC2238934

[B65] Wood V Gwilliam R Rajandream MA Lyne M Lyne R Stewart A Sgouros J Peat N Hayles J Baker S The genome sequence of Schizosaccharomyces pombe Nature 2002 415 6874 871 880 10.1038/nature724 11859360

[B66] Goffeau A Barrell BG Bussey H Davis RW Dujon B Feldmann H Galibert F Hoheisel JD Jacq C Johnston M Life with 6000 genes Science 1996 274 5287 546 547 10.1126/science.274.5287.546 8849441

