# Supplementary material for: Genome-wide in silico screen for CCCH-type zinc finger proteins of Trypanosoma brucei, Trypanosoma cruzi and Leishmania major
Source: BMC Genomics. 2010 May 5;11:283. doi: 10.1186/1471-2164-11-283 (PMC2873481; doi:10.1186/1471-2164-11-283)
Supplement: Additional file 1 — Figure S1: equence logos of the C-X7-C-X5-C-X3-H and C-X8-C-X5-C-X3-H zinc fingers of Tb, Tc and Lm. Only CCCH motifs that were recognized by at least either Smart, Pfam or InterPro are included. Tc CCCH motifs present more than once due to allelic variants were not included. The sequence logos shown here were used to create the combined Tritryp sequence logos shown in Figure 1. [file 1471-2164-11-283-S1.PDF]

FIGURE S1

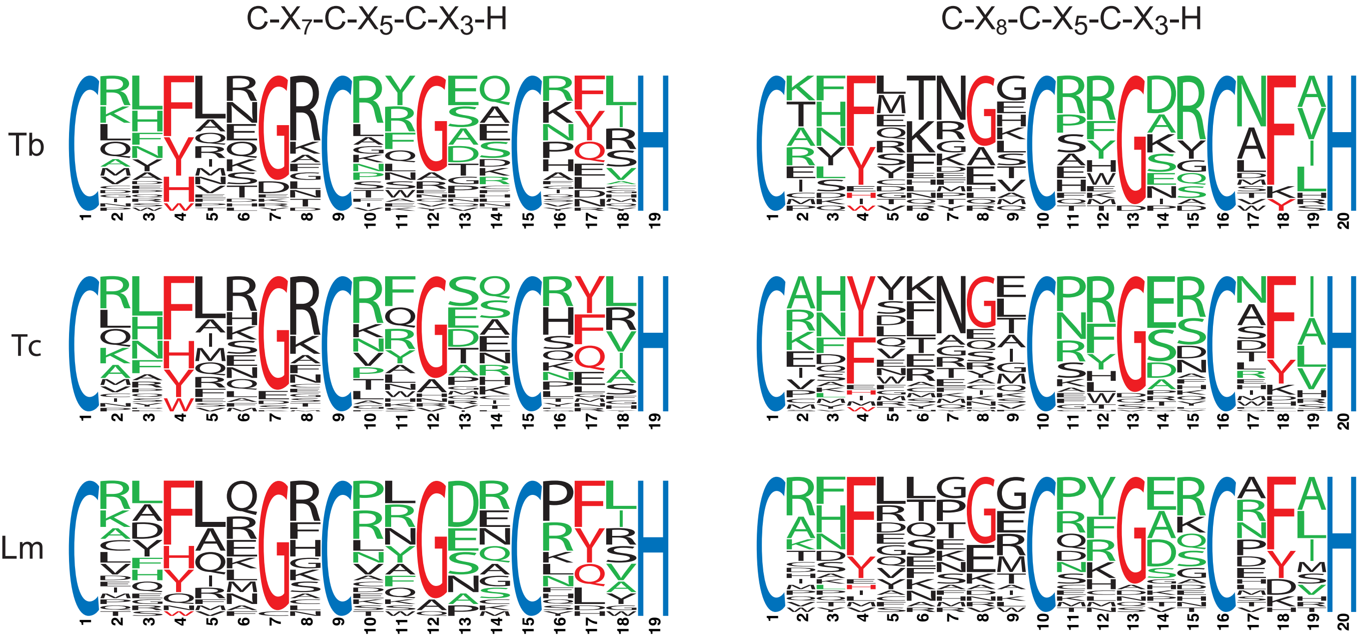

Figure S1: **Sequence logos of the C-X7-C-X5-C-X3-H and C-X8-C-X5-C-X3-H zinc fingers of Tb, Tc and Lm.** Sequence logos for all C-X_7-C-X_5-C-X_3-H and C-X_8-C-X_5-C-X_3-H zinc finger motifs identified in *T. brucei* (Tb), *T. cruzi* (Tc) and *L. major* (Lm) that were recognized by at least either SMART (Sm00356), Pfam (FP00642) or Inter-pro (IPR000571).Conserved amino acids that were used for the evaluation of unknown CCCH motifs are shown in red (very conserved, used in first filtering) and green (conserved, used in second filtering). The sequence logos were created using WEBLOGO [48].
