# Supplementary material for: Genome-wide in silico screen for CCCH-type zinc finger proteins of Trypanosoma brucei, Trypanosoma cruzi and Leishmania major
Source: BMC Genomics. 2010 May 5;11:283. doi: 10.1186/1471-2164-11-283 (PMC2873481; doi:10.1186/1471-2164-11-283)
Supplement: Additional file 2 — Table S1: List of the Tritryp CCCH proteins. List of the Tritryp CCCH proteins, including molecular weight, class of the CCCH motif and redundant genes. [file 1471-2164-11-283-S2.DOC]

Table S1: Complete list of CCCH proteins in the Tritryps.

|  | **ID** | | | **Class**  **(C-X_Y_-C-X_Z_-C-X_3_-H)** | | | | | | **Molecular weight** | | |  |
| --- | --- | --- | --- | --- | --- | --- | --- | --- | --- | --- | --- | --- | --- |
| **Protein** | **Tb** | **Tc** | **Lm** | **Tb** | | **Tc** | | **Lm** | | **Tb** | **Tc** | **Lm** | no of CCCH motifs |
|  |  |  |  | **Y** | **Z** | **Y** | **Z** | **Y** | **Z** |  |  |  |  |
|  |  |  |  |  |  |  |  |  |  |  |  |  |  |
| CPSF30 | Tb11.01.4600 | Tc00.1047053510219.30 | LmjF09.0720 | 7 | 5 | 7 | 5 | 7 | 5 | 31.6 | 31.3 | 36.4 | 5 |
|  |  | Tc00.1047053511555.40 |  | 7 | 5 | 7 | 5 | 7 | 5 |  | 31.3 |  |  |
|  |  |  |  | 8 | 4 | 8 | 4 | 8 | 4 |  |  |  |  |
|  |  |  |  | 7 | 5 | 7 | 5 | 7 | 5 |  |  |  |  |
|  |  |  |  | 7 | 5 | 7 | 5 | 7 | 5 |  |  |  |  |
| ZC3H32 | Tb927.10.5250 | Tc00.1047053503795.10*^1^ | LmjF36.0840 | 7 | 5 | sequene  missing | | 7 | 5 | 72.1 | 52.3 | 84.8 | 3 (Lm, Tb) |
|  |  | Tc00.1047053506679.10*^1^ |  | 9 | 5 | 9 | 5 | 9 | 5 |  | 49.8 |  | 2 (Tc) |
|  |  |  |  | 7 | 5 | 7 | 5 | 7 | 5 |  |  |  |  |
| ZC3H31 | Tb927.10.5150 | Tc00.1047053506009.10 | LmjF36.0730 | 8 | 5 | 8 | 5 | 8 | 5 | 57.5 | 50.3 | 60.9 | 3 |
|  |  | Tc00.1047053510295.59*^2^ |  | 8 | 5 | 8 | 5 | 8 | 5 |  |  |  |  |
|  |  | Tc00.1047053510297.10*^2^ |  | 8 | 5 | 8 | 5 | 8 | 5 |  |  |  |  |
| hypothetical | absent | absent | LmjF36.0740 | 8 | 5 | 8 | 5 | 8 | 5 |  |  | 61.1 | 3 |
| ZC3H30 | Tb927.10.1540 | Tc00.1047053506977.110*^9^ | LmjF21.0770 | 7 | 4 | pseudo | | 7 | 4 | 60.2 |  | 52.9 | 2 |
|  |  |  |  | 7 | 5 | pseudo | | 7 | 5 |  |  |  |  |
| ZC3H21 | Tb927.7.2670 | Tc00.1047053511817.10 | LmjF22.0740 | 7 | 5 | 7 | 5 | 7 | 5 | 49.7 | 48.7 | 61.9 | 2 |
|  |  | Tc00.1047053506859.230 |  | 7 | 5 | 7 | 5 | 7 | 5 |  | 48.7 |  |  |
| ZC3H27 | Tb09.160.2090 | Tc00.1047053504797.110 | LmjF01.0210 | 7 | 5 | 7 | 5 | 7 | 5 | 87.96 | 91.2 | 95.9 | 2 |
|  |  | Tc00.1047053511577.30 |  | 7 | 5 | 7 | 5 | 7 | 6 |  | 95.5 |  |  |
| ZC3H18 | Tb927.7.2140 | Tc00.1047053511807.160 | LmjF22.0160 | 7 | 5 | 7 | 5 | 7 | 5 | 40.87 | 39.4 | 43.9 | 2 |
|  | (Tb07.11L3.90) |  |  | 7 | 5 | 7 | 5 | 7 | 5 |  |  |  |  |
| ZC3H20 | Tb927.7.2660 | Tc00.1047053503567.9 | absent | 7 | 5 | 7 | 5 | absent | | 43.76 | 47.8 |  | 2 |
|  |  | Tc00.1047053506859.204 |  | 7 | 5 | 7 | 5 |  |  |  | 47.8 |  |  |
| conserved | absent | Tc00.1047053506883.120 | LmjF35.5200 | absent | | 8 | 5 | 8 | 5 |  | 22.7 | 28.3 | 2 |
|  |  | Tc00.1047053510427.10 |  |  |  | 7 | 4 | 7 | 4 |  | 22.6 |  |  |
| ZC3H29 | Tb09.211.1130 | Tc00.1047053510729.210 | absent | 7 | 5 | 7 | 5 | absent | | 37.75 | 35 |  | 2 |
|  |  | Tc00.1047053506885.204 |  | 8 | 4 | 8 | 4 |  |  |  | 35 |  |  |
| conserved | absent | Tc00.1047053509395.40 | LmjF02.0100*^8^ | absent | | 7 | 5 | pseudo | |  | 103.3 | 96.4 | 2 |
|  |  | Tc00.1047053411427.10*^6^ |  |  |  | 8 | 5 |  |  |  | 25.3 |  |  |
| hypothetical | absent | absent | LmjF04.0040 | absent | | absent | | 8 | 5 |  |  | 46.9 | 2 |
|  |  |  |  |  |  |  |  | 7 | 5 |  |  |  |  |
| ZC3H22 | Tb927.7.2680 | Tc00.1047053506859.240 | LmjF22.0750 | 7 | 5 | 7 | 5 | 7 | 5 | 76.2 | 56.8 | 119.2 | 2 |
|  |  | Tc00.1047053511817.20 |  | 8 | 5 | 8 | 5 | 8 | 5 |  | 68.2 |  |  |
| ZC3H46 | Tb11.01.8270 | Tc00.1047053507089.30 | absent | 8 | 5 | 8 | 5 | absent | | 56.45 | 49.8 |  | 2 |
|  |  | Tc00.1047053504085.70 |  | 7 | 5 | 7 | 5 |  |  |  | 48.7 |  |  |
| conserved | absent | Tc00.1047053511671.130 | LmjF28.2950 | absent | | 7 | 5 | 7 | 5 |  | 33.4 | 36.2 | 2 |
|  |  | Tc00.1047053508831.66 |  |  |  | 8 | 5 | 11 | 5 |  | 33.5 |  |  |
| ZC3H43 | Tb11.02.5350 | Tc00.1047053511151.20 | LmjF28.0300 | 7 | 6 | 7 | 6 | 7 | 5 | 68.21 | 63 | 70.8 | 2 |
|  |  | Tc00.1047053508241.90 |  | 8 | 5 | 8 | 5 | 8 | 5 |  | 63 |  |  |
| U2af35 | Tb927.10.3200 | Tc00.1047053510943.60 | LmjF03.0190 | 8 | 5 | 8 | 5 | 8 | 5 | 29.12 | 27.4 | 25.2 | 2 |
|  |  | Tc00.1047053503577.20 |  | 7/8 | 5 | 7/8 | 5 | 7/8 | 5 |  | 27.4 |  |  |
| conserved | absent | Tc00.1047053511807.130 | LmjF22.0130*^7^ | absent | | 8 | 5 | pseudo | |  | 48.7 | 49.4 | 2 (Tc) |
|  |  |  |  |  |  | 8 | 5 |  |  |  |  |  | 1 (Lm) |
| ZC3H37/38 | Tb927.10.12780 | Tc00.1047053507831.20 | absent | 10 | 5 | 10 | 5 | absent | | 40.4 | 31.5 |  | 2 |
|  | Tb927.10.12800 | Tc00.1047053511267.20 |  | 8 | 5 | 8 | 5 |  |  | 27.6 | 30.9 |  |  |
|  |  | pseudo (seq. error) |  |  |  |  |  |  |  |  |  |  |  |
|  |  | Tc00.1047053511263.50 |  |  |  |  |  |  |  | 43.7 |  |  |  |
| ZC3H36 | Tb927.10.12760 | Tc00.1047053507831.30 | absent | 10 | 5 | 10 | 5 | absent | | 34.9 | 53.9 |  | 2 |
|  |  | Tc00.1047053511263.40 |  | 8 | 5 | 8 | 5 |  |  |  | 54 |  |  |
| ZC3H35 | Tb927.10.12740 | Tc00.1047053511263.30 | LmjF18.1350 | 10 | 5 | 10 | 5 | 10 | 5 | 51.3 | 62.1 | 66.3 | 2 |
|  |  | Tc00.1047053507831.40 |  | 8 | 5 | 8 | 5 | 8 | 5 |  | 62.2 |  |  |
| conserved | absent | Tc00.1047053511267.24 | LmjF18.1340 | absent | | 10 | 5 | 10 | 5 |  | 82.1 | 77.7 | 2 |
|  |  | Tc00.1047053509805.230 |  |  |  | 8 | 5 | 8 | 5 |  | 62.1 |  |  |
| conserved | Tb927.10.8850 | Tc00.1047053511039.39 | LmjF36.5940 | 5 | 5 | 5 | 5 | 5 | 5 | 65.9 | 65.6 | 68.3 | 1 |
|  |  | Tc00.1047053509791.90 |  |  |  |  |  |  |  |  | 65.6 |  |  |
| conserved | Tb09.211.1720 | Tc00.1047053509551.60 | LmjF35.4450 | 7 | 4 | 7 | 4 | 7 | 4 | 13.7 | 26.2 | 29 | 1 |
|  |  | Tc00.1047053507007.77 |  |  |  |  |  |  |  |  | 25.6 |  |  |
| conserved | Tb11.22.0004*^3^ | Tc00.1047053508271.4*^4^ | LmjF27.1690 | 7 | 4 | 7 | 4 | 7 | 4 | 62.5 | 27.8 | 87.5 | 1 |
|  |  | Tc00.1047053506127.20 |  |  |  |  |  |  |  |  | 63 |  |  |
| hypothetical | absent | absent | LmjF10.0483 | absent | | absent | | 7 | 4 |  |  | 60.3 | 1 |
| conserved | Tb09.211.4670 | Tc00.1047053506999.120 | LmjF35.1510 | 7 | 4 | 7 | 4 | 7 | 5 | 59.1 | 56.2 | 64.4 | 1 |
|  |  | Tc00.1047053510759.100 |  |  |  |  |  |  |  |  | 56.2 |  |  |
| conserved | Tb927.6.4960 | Tc00.1047053506945.210 | LmjF30.3570 | 7 | 4 | 7 | 4 | 8 | 4 | 35 | 34.6 | 29.6 | 1 |
| ZC3H34 | Tb927.10.12330 | Tc00.1047053507787.140 | LmjF31.0080 | 7 | 5 | 7 | 5 | 7 | 5 | 22.4 | 22.4 | 25.7 | 1 |
|  |  | Tc00.1047053507625.70 |  |  |  |  |  |  |  |  | 22.4 |  |  |
| ZC3H19 | Tb927.7.2580 | Tc00.1047053506859.80 | LmjF22.0660 | 7 | 5 | 7 | 5 | 7 | 5 | 68.4 | 74.4 | 113.6 | 1 |
|  |  | Tc00.1047053511815.50 |  |  |  |  |  |  |  |  | 73.4 |  |  |
| ZC3H39 | Tb927.10.14930 | Tc00.1047053508895.50 | LmjF19.0300 | 7 | 5 | 7 | 5 | 7 | 5 | 31.5 | 31 | 35 | 1 |
| CSBPA |  | Tc00.1047053506211.70 |  |  |  |  |  |  |  |  | 31.1 |  |  |
| ZC3H8 | Tb927.3.5250 | Tc00.1047053510143.120 | LmjF29.0420 | 7 | 5 | 7 | 5 | 7 | 5 | 61.2 | 55.6 | 41.3 | 1 |
|  |  | Tc00.1047053508409.310 |  |  |  |  |  |  |  |  | 55.4 |  |  |
| ZC3H5 | Tb927.3.740 | Tc00.1047053507775.10 | LmjF27.0150 | 7 | 5 | 7 | 5 | 7 | 5 | 25.5 | 27.3 | 19.4 | 1 |
|  |  | Tc00.1047053511867.10 |  |  |  |  |  |  |  |  | 27.2 |  |  |
| ZC3H6 | Tb927.3.790 | Tc00.1047053511867.60 | LmjF27.0200 | 7 | 5 | 7 | 5 | 7 | 5 | 38.7 | 39.5 | 40.2 | 1 |
| ZC3H9 | Tb927.4.1310 | Tc00.1047053508693.40 | LmjF34.3460 | 7 | 5 | 7 | 5 | 7 | 5 | 47.4 | 54 | 53.6 | 1 |
| FIP1 | Tb927.5.4320 | Tc00.1047053510351.80 | LmjF05.0190 | 7 | 5 | 7 | 5 | 7 | 5 | 31 | 31.2 | 38.5 | 1 |
|  |  | Tc00.1047053507601.80 |  |  |  |  |  |  |  |  | 31.4 |  |  |
| ZC3H23 | Tb927.7.4980 | Tc00.1047053508175.350 | LmjF06.0380 | 7 | 5 | 7 | 5 | 7 | 5 | 43.4 | 45.4 | 44.6 | 1 |
|  |  | Tc00.1047053509149.20 |  |  |  |  |  |  |  |  | 44.8 |  |  |
| ZC3H40 | Tb927.10.14950 | Tc00.1047053506211.60 | absent | 7 | 5 | 7 | 5 | absent | | 46.2 | 60.9 |  | 1 |
| CSBPB |  | Tc00.1047053508895.60 |  |  |  |  |  |  |  |  | 51.6 |  |  |
| ZC3H45 | Tb11.01.0090 | Tc00.1047053506931.4 | LmjF28.1405^*5)^ | 7 | 5 | 7 | 5 | 7 | 5 | 22.9 | 24 | 41.4 | 1 |
|  |  | Tc00.1047053510131.44 |  |  |  |  |  |  |  |  | 22.6 |  |  |
| ZC3H15 | Tb927.6.4720 | absent | LmjF30.3370*^3^ | 7 | 5 | absent | | 7 | 5 | 21.92 |  | 68.4 | 1 |
| hypothetical | absent | absent | LmjF34.1240 | absent | | absent | | 7 | 5 |  |  | 60.2 | 1 |
| ZC3H28 | Tb09.211.1070 | Tc00.1047053506885.200 | LmjF35.4950 | 7 | 5 | 7 | 5 | 8 | 5 | 114.78 | 113.9 | 107.3 | 1 |
|  |  | Tc00.1047053510729.220 |  |  |  |  |  |  |  |  | 113.4 |  |  |
| ZC3H41 | Tb11.46.0009 | Tc00.1047053508355.330 | LmjF27.1300 | 7 | 5 | 7 | 5 | 8 | 5 | 58.47 | 59.1 | 59.9 | 1 |
|  |  | Tc00.1047053508357.9 |  |  |  |  |  |  |  |  | 29.8 |  |  |
| conserved | Tb11.02.1470 | Tc00.1047053509229.90 | LmjF13.0880 | 8 | 4 | 8 | 4 | 8 | 4 | 78.04 | 85.3 | 76.5 | 1 |
|  |  | Tc00.1047053506733.140 |  |  |  |  |  |  |  |  | 85 |  |  |
| ZFP2 | Tb11.01.6590 | Tc00.1047053401469.10 | LmjF32.1740 | 8 | 5 | 8 | 5 | 8 | 5 | 15.77 | 20 | 14.4 | 1 |
|  |  | Tc00.1047053503989.10 |  |  |  |  |  |  |  |  | 20.1 |  |  |
| ZC3H44 | Tb11.02.5760 | Tc00.1047053506933.50 | LmjF28.0830 | 8 | 5 | 8 | 5 | 8 | 5 | 48.19 | 48.2 | 59.8 | 1 |
| ZC3H7 | Tb927.3.1340 | Tc00.1047053509233.210 | LmjF25.1220 | 8 | 5 | 8 | 5 | 8 | 5 | 66.66 | 62.5 | 65.8 | 1 |
| ZFP3 | Tb927.3.720 | Tc00.1047053509231.39 | LmjF27.0130^*3^ | 8 | 5 | 8 | 5 | 8 | 5 | 14.17 | 17.7 | 16.8 | 1 |
|  |  | Tc00.1047053509719.69 |  |  |  |  |  |  |  |  | 14.7 |  |  |
| ZC3H12 | Tb927.5.1570 | Tc00.1047053506739.99 | LmjF15.0140 | 8 | 5 | 8 | 5 | 8 | 5 | 18.78 | 19.5 | 23.8 | 1 |
|  |  | Tc00.1047053510819.119 |  |  |  |  |  |  |  |  | 19.5 |  |  |
| ZC3H11 | Tb927.5.810 | Tc00.1047053507305.40 | LmjF35.1040 | 8 | 5 | 8 | 5 | 8 | 5 | 39.56 | 29.5 | 36.4 | 1 |
|  |  | Tc00.1047053504929.5 |  |  |  |  |  |  |  |  | 29.6 |  |  |
| hypothetical | absent | absent | LmjF35.1020 | 8 | 5 | 8 | 5 | 8 | 5 |  |  | 45.3 | 1 |
| ZFP1 | Tb927.6.3490 | Tc00.1047053511511.6^3*^ | LmjF30.2200 | 8 | 5 | 8 | 5 | 8 | 5 | 11.5 | 12.8 | 18 | 1 |
| ZFP1B |  | Tc00.1047053511511.3 |  |  |  |  |  |  |  |  | 14.2 |  |  |
| ZC3H24 | Tb927.8.4020 | Tc00.1047053503897.150 | LmjF10.0640 | 8 | 5 | 8 | 5 | 8 | 5 | 25.5 | 29.1 | 25.7 | 1 |
| ZC3H25 | Tb927.8.4070 | Tc00.1047053509561.39 |  |  |  |  |  |  |  | 25.5 | 28.9 |  |  |
| ZC3H26 | Tb927.8.4120 |  |  |  |  |  |  |  |  | 25.5 |  |  |  |
| ZC3H17 | Tb927.7.930 | Tc00.1047053508215.10 | LmjF26.1010 | 8 | 5 | 8 | 5 | 8 | 5 | 52.4 | 41.7 | 45.9 | 1 |
|  |  | Tc00.1047053508879.10 |  |  |  |  |  |  |  |  | 54.3 |  |  |
| ZC3H13 | Tb927.5.1580 | absent | LmjF15.0160 | 8 | 5 | absent | | 8 | 5 | 60.31 |  | 85.7 | 1 |
| ZC3H14 | Tb927.6.4050 | Tc00.1047053511521.20 | LmjF30.2780^*5)^ | 8 | 5 | 8 | 5 | 8 | 5 | 29.8 | 29.5 | 36.3 | 1 |
|  |  | Tc00.1047053511735.40 |  |  |  |  |  |  |  |  | 29.6 |  |  |
|  |  | Tc00.1047053511735.88 |  |  |  |  |  |  |  |  | 19.8 |  |  |
| DNAJ / TTP | absent | Tc00.1047053503453.20 | LmjF35.0230 | absent | | 8 | 5 | 8 | 5 |  | 39.2 | 39.7 | 1 |
|  |  | Tc00.1047053511467.4*^4^ |  |  |  |  |  |  |  |  | 28 |  |  |
| hypothetical | absent | absent | LmjF32.0030 | absent | | absent | | 8 | 5 |  |  | 40.4 | 1 |
| hypothetical | absent | absent | LmjF32.0040 | absent | | absent | | 8 | 5 |  |  | 37 | 1 |
| ubiquitin ligase | Tb927.8.1590 | Tc00.1047053508971.50 | LmjF07.0280 | 10 | 5 | 10 | 5 | 10 | 5 | 470.8 | 456.9 | 669.4 | 1 |
| WW/Rsp5/WWP | absent | absent | LmjF36.5980 | absent | | absent | | 11 | 5 |  |  | 54.5 | 1 |

| *1) Both Tc proteins lack the N-terminal CCCH finger (7/5) due to N-terminal truncations (do not start with start codons). Tc00.1047053503795 also lacks the 9/5 finger due to a minor differences at the (truncated) N-terminus in comparison to Tc00.1047053506679.10. |
| --- |
| *2) These two genes present fragments of the same gene (orthologue to Tc00.1047053506009.10), sequenced on two different contigs. |
| *3) wrong start codon annoted in GeneDB |
| *4) partial gene |
| *5) The orthologue assignment was done by genomic syntheny. |
| *6) This gene codes for a partial protein that contains only the C-terminus, including the second (C-X_8_-C-X_5_-C-X_3_-H) CCCH motif. |
| *7) This gene is declared a pseudogene, it is a real gene in Lb. The first CCCH motif is only present in Tc and Lb, but absent from the Lm pseudogene. |
| *8) This gene is declared a pseudogene in Lm but not in Li. |
| *9) This gene is declared a pseudogene. |
