# Supplementary material for: Genome-wide in silico screen for CCCH-type zinc finger proteins of Trypanosoma brucei, Trypanosoma cruzi and Leishmania major
Source: BMC Genomics. 2010 May 5;11:283. doi: 10.1186/1471-2164-11-283 (PMC2873481; doi:10.1186/1471-2164-11-283)
Supplement: Additional file 3 — Figure S2: Alignment of the putative TbMex67 with homologues of other organisms. A) Alignment of S. cerevisiae Mex67 with T. brucei Tb11.22.0004 B) Alignment of region of greatest identity between S. cerevisiae Mex67, Tb11.22.0004 and D. melanogaster NXF1. [file 1471-2164-11-283-S3.PDF]

Figure S2

A

CCCH motif

```
Sc          1 MSGFHNVGNINMMAQQQMQQNRIKISVRNWQNATMNDLINFISRNARVAV     50
                                    :....|..::.:....:.|  .|.:....
Tb          1 ----------------------MPNPYRKNKDTSRIPCVYF-KRGSCANK     27

Sc         51 Y-DA-HVEGPLVIGYVNSKAEAESLMKWNGVRFAGSNLKFELLDNNGASA     98
              : ..  ||.|...:|:   ..::|.       |.   .|:||...:|
Tb         28 HCPFLHVRGGEKVGH--EPGKAP------GD--VGTNLLCTML-------     60

Sc         99 GTSDTISFLRGVLLKR-YDPQTKLLNLGALHSDPELIQKGVFSSISTQSK    147
                  .:.|.:   ..:|  |:.:|.:|||..:...|:|   ..|..||:.:::
Tb         61 ----KLVFEK--QQQRVYNAETGVLNLTQFKEIPDL--TDVAGSINFNTQ    102

Sc        148 MFPAMMKLASTEKSLIV---ESVNLADNQLKDIS--AISTLAQTFP-NLK    191
              .|   ...|.||.|||||    .:::|..|.:..:|   |::......:. .|:
Tb        103 AF--CQALCSTIKSLIVPPPSAIQLKGNGILSVSHLAMQLEKADLHLTLR    150

Sc        192 NLCLANNQIFRFRSLEVWKNKFKDLRELLMTNNPITTDKLYRTEMLRLFP    241
              .:.|..|.|....||:..| ||.:|||:::.:|||.....|||.:.:|.|
Tb        151 AISLEANLIKSVESLQELK-KFTNLREIVLRDNPIANRSDYRTTIKKLMP    199

Sc        242 KLVVLD--N-----VIVRDEQKLQTVYSLP----MKIQQF--F--FENDA    276
              .|:.||  :      :.:...|...|.||..     :::.|.  :   :|.|
Tb        200 SLIGLDGESICVPPLSLPWPQFSPTGYSEAQKHVLQFIQCGLLNPLEGD-    248

Sc        277 LGQSSTDFATNFL-NLWDNNREQLLNLYSPQSQFSVSVDSTIPPSTVTDS    325
               |.|.:.:::.: :|:..|....|:|.||   :   |:|  ||  |..|:..:
Tb        249 -GASGVAHGVDAVSDLYAANAVLTLSLSSP--E--VAV-ST-PMRTMNGA    291

Sc        326 DQTPAFGYYMSSSRN-I----SKVSSEKS----IQQRL-SIGQESINSIF    365
NTF2          ..:|.   ..:..|: :     .::.|..:     :::.: ::|:..:.|..
Tb        292 AGVPQ---QRNVIRDIVSLRLKQTESNHNLLHGVKSTVVALGRTKVCSQM    338

Sc        366 K--TLPKT--KHHLQEQPNEYSMETISY-----PQING--F-VITLHG--    401
              :  ..|||  .||...:.........||      |....   . ::||||
Tb        339 EHWLYPKTFRVHHYLHSSASTVFLDNSYLTGPSPTAMKVPVTIVTLHGTM    388

Sc        402 FFEETGKPELESNKKTGKNNYQKNRRYNHGYNSTSNNKLSKKSFDRT-WV    450
              .:..||.|     ...|.....:.. ||      |   ....||..:.|.. |:
Tb        389 TWNNTGVP----GDATTIGPMTI-RR-----N--FTRVLSVTQGDAGRWL    426

Sc        451 IVPMNNSVIIASDLLTVRAYSTGAWKTA-S-IAIAQPPQQQAS--VL-PQ    495
              |      :   :|:::.....:|:..:: | .::.:..|.:..   :| |:
Tb        427 I------T---NDMVSLYLTPSGSSASSKSGNGVPETTQLSECRILLDPR    467

Sc        496 VASMNPNITTPPQ--PQPSVVP----GGMSIP---------GAPQGAMVM    530
              .........:.:.:  |..::.     .|....          |.|..|..:
Tb        468 SDRSRAEVLSRKKDVPVEVIMALCQHVGNDAELFTVLDDIRGVPLSAFEH    517

Sc        531 APTLQLPPDVQSRLNPVQL-ELLNKLHLETKLNAEYTFMLAEQS-NWNYE    578
              ...|. .:.:...     :|: .|:|.:.:..: || .. ||..:: ||:..
Tb        518 CANLA-GENIMES---IQMCRLVNLFGMAPQ-NA-LE-MLRHNNGNWSDT    560
TAP-C
Sc        579 VAIKGFQSSMNGIPREAFVQF    599
              ||..:..:.:.::|:
Tb        561 VAAVAATAPVAAVPQ------    575
```

B

```
Dm    SGIPLVAIDDAFKEKMKVTMAKRYNIQTKALDLSRFHADPDLKQ--VFCPLFRQNVMGAA  245
                  +  ++  + KRY+ QTK L+L   H+DP+L Q  VF  +  Q+ M  A
Sc    ASAGTSDTISFLRGVLL----KRYDPQTKLLNLGALHSDPELIQKGVFSSISTQSKMFPA  151
         GT+   + L+ V      + Y+ +T +LNL      P+L    V  SI+  ++ F
Tb    GDVGTNLLCTMLKLVFEKQQQRVYNAETGVLNLTQFKEIPDLTD--VAGSINFNTQAF--  104


Dm    IDIMCDNIPDL-----EALNLNDNSISSMEAFKG---VEKRLPNLKILYLGDNKIPSLAH  297
      +  +      L     E++NL DN +  + A      + +  PNLK L L +N+I
Sc    MMKLASTEKSLIV---ESVNLADNQLKDISAIST---LAQTFPNLKNLCLANNQIFRFRS  205
         L ST KSLIV    ++ L  N +  +S ++     A     L+ + L  N I    S
Tb    CQALCSTIKSLIVPPPSAIQLKGNGILSVSHLAMQLEKADLHLTLRAISLEANLIKSVES  164


Dm    LVVLRN--LSILELVLKNNPCRSRYKDSQQFISEVRRKFPKLVKLDG  342
      L V +N    + EL++ NNP +      + + +E+ R FPKLV LD
Sc    LEVWKNKFKDLRELLMTNNPITT----DKLYRTEMLRLFPKLVVLDN  248
      L+  K KF +LRE+++ +NPI         YRT + +L P L+ LD
Tb    LQELK-KFTNLREIVLRDNPIAN----RSDYRTTIKKLMPSLIGLDG  206
```

Figure S2: **Alignment of the putative TbMex67 with homologues of other organisms.**
A) Alignment of *S. cerevisiae* (Sc) Mex67 and *Trypanosoma brucei* (Tb) Tb11.22.0004 produced by Needle at http://www.ebi.ac.uk/Tools/emboss/align/index.html and using the blosum 40 matrix. There is 20.1% identity over the whole length of the two polypeptides. Domains recognized in the yeast Mex67 are indicated. The CCCH motif (only present in Tb Mex67) is framed and shown in the same colours as in Figure 1.
B) Alignments of the region of greatest identity between *S. cerevisiae* (Sc) Mex67 and *Trypanosoma brucei* (Tb) Tb11.22.0004 produced using NCBIblast2. A similar alignment of Mex67 and *Drosophila melanogaster* (Dm) NXF1 is shown for comparison.
