# Supplementary material for: Genome-wide in silico screen for CCCH-type zinc finger proteins of Trypanosoma brucei, Trypanosoma cruzi and Leishmania major
Source: BMC Genomics. 2010 May 5;11:283. doi: 10.1186/1471-2164-11-283 (PMC2873481; doi:10.1186/1471-2164-11-283)
Supplement: Additional file 4 — Figure S3: Alignment of the Leishmania 3'-5' exoribonuclease (LmjF34.1240) with the homologous proteins of Mus musculus. [file 1471-2164-11-283-S4.PDF]

FIGURE S3

```
M. musculus         MSRQNLVALTVTTLLGVAMGGFVLWKGIQRRWSKTSRVMQQQPQQPQQPQQPQPQPQPQP 60
LmjF34.1240         ------------------------------------------------------------

M. musculus         QPQPEHPQPQQQVPGGREWPPPEDDQLPFGALRAPRASWEERILQAEVVTVSQEAEWNQI 120
LmjF34.1240         -----------------------------------------------------------M 1
                                                                               :
```

3'-5' exoribonuclease domain

```
M. musculus         QPFLKRELEDFPVLGIDCEWVNLEGKASPLSLLQMASPSGFCALVRLPRLIYGGRTLPRT 180
LmjF34.1240         MQTLRQQPAHLKIMGMDSEWC----RNLPLAVVQFATSS-HCFVLHIS--FFDGRTLPAA 54
                       *:::  .: ::*:*.**     :  **:::*:*:.* .* ::::.  ::.***** :

M. musculus         LLDILADGAILKVGVGCSEDANKLLQDYGLIVRGCLDLRYLAMKQGNNILCNGLSLKSLA 240
LmjF34.1240         VKEALCDPSIIKCGVGVSGDVSRLQKEQNITIQSVLDVAQYSALFGLHQGAQSN-LKVLA 113
                    : : *.* :*:* *** * *..:* :: .: ::. **:   :   * :  .:.  ** **

M. musculus         ETILNFPLDKSLLLRCSNWDAENLTEDQVTYAARDAQISVALFLHLLGYPFSRDSYEEES 300
LmjF34.1240         KSVANLSIEKDKMITRSNWELP-LSDSRVNYAAEDALASYLVGRAVMLKASEVTDMSADT 172
                    ::: *:.::*. ::  ***:   *::.:*.***.**  *  :   ::  . .  . . ::

M. musculus         TDQINWQKALERCRNMVDIPFRSKGLGRLVEEVN----------GEALESQLKPRNRKAK 350
LmjF34.1240         FDAVQWLQRTSSKAAMELKKLQRETSKSEAEKRKKACPASKISGGDALLAQFRSGTKVRV 232
                     * ::* :  .    *    :: :     .*: :          *:** :*::. .:

M. musculus         TDR---MVPGNNQGRDPRKHKRKPLGVGYSARKSPLYDNCFLQAPDGQPLCTCDRRKAQW 407
LmjF34.1240         LDRNGNFIFECSSGRGKFYVMEKNLAVITKHAKSDPRKALEIQLLFDP----KVKVRLCM 288
                     **   ::   ..**.     .* *.*  .  **   .   :*   .       : :
```

CCCH motif (Lm)

```
M. musculus         YLDKGIGELVSKEPFVVRLQFEPAGRPESPGDYYLMVKENLCVVCGKTDTYIRKNIIPHE 467
LmjF34.1240         YQALGFCELQDQCPFAH-----GASELQADAAALVDSQIPSCACCLGTKGLLRHAITPPS 343
                    *   *: ** .: **.       *.. :: .   :  :   *. *  *.  :*: * * .

M. musculus         YRKHFPIEMKDHNSHDVLLLCTSCH-AISNYYDNHLKQQLAKEFQAP-----IGSEEGLR 521
LmjF34.1240         FRKFMPPPQRQALDDDYLPVCKQCNSTLRLYYDEEMKRCYTQAEESSTMVCDVKVAAKCT 403
                    :**.:*   ::  ..* * :*..*: ::  ***:.:*:  ::  ::.     :

M. musculus         LLEDLERRQVRSGARALLNAESLPAHRKEELLHALREFYNTDIITEEMLHEAASLETRIY 581
LmjF34.1240         LYARLLLDADKLAKIPANRREELQQFVKQNWKSTLFEEFNSGFEIHEPVVREAAFLERLG 463
                    *   *     : .  .  . *.*  . *::   :* * :*:.:  .* : . *::  *:

M. musculus         NESYIPHGLKVVQRHTEGG-LRSLMQLESRWRQHLLDSIQPKHLPQQWSVDHNHQKLLRK 640
LmjF34.1240         KIVPGDMRAKVTMAVLVGDDEEKAQQFNKRWRDYCFSTCGLMEKKSNHMSYNAWKAYRAH 523
                    :        **.     *.  ..  *::.***:: :.:    .  .:    :  :    :

M. musculus         YGDDLPIKLS----- 650
LmjF34.1240         NGEPQADDEEDGTPQ 538
                     *:  . . .
```

Figure S3 Alignment of LmjF34.1240 with the 3'-5' exoribonucleases of *M. musculus* (BAE27515).
The 3'-5' exoribonuclease domain is shadowed in grey. The CCCH motif of the Leishmania protein is framed in red and colour-coded as in Figure 1.
